# ^1^H-NMR Metabolomics Study after Foliar and Endo-Therapy Treatments of *Xylella fastidiosa* subsp. *pauca* Infected Olive Trees: Medium Time Monitoring of Field Experiments

**DOI:** 10.3390/plants12101946

**Published:** 2023-05-10

**Authors:** Mudassar Hussain, Chiara Roberta Girelli, Dimitri Verweire, Michael C. Oehl, Maier S. Avendaño, Marco Scortichini, Francesco Paolo Fanizzi

**Affiliations:** 1 Department of Biological and Environmental Sciences and Technologies, University of Salento, Prov.le Lecce-Monteroni, 73100 Lecce, Italy; mudassar.hussain@unisalento.it; 2 Invaio Sciences, Cambridge, MA 02138, USA; dverweire@invaio.com (D.V.); moehl@invaio.com (M.C.O.); mavendano@invaio.com (M.S.A.); 3 Council for Agricultural Research and Agricultural Economic Analyses (CREA), Research Centre for Olive, Fruit and Citrus Crops, Via di Fioranello, 52, 00134 Roma, Italy; marco.scortichini@crea.gov.it

**Keywords:** endo-therapy, foliar spray, ^1^H-NMR, metabolomics, *Xylella fastidiosa*, olive trees, Dentamet, field experiments, Olive Quick Decline Syndrome

## Abstract

Here we report the medium-term effects of foliar spray and endo-therapy treatments with different doses of a Cu/Zn citric acid biocomplex (Dentamet^®^) in *Xylella fastidiosa* infected olive trees of Salento, Apulia region (South-east Italy). Leaf extract samples from field-treated 150 years old olive trees cvs Ogliarola salentina and Cellina di Nardò were studied by ^1^H NMR-based metabolomics. The result of different applications of Dentamet^®^ endo-therapy after 60, 120 and 180 days in comparison with traditional foliar spray treatment and water injection as a control have been investigated. The metabolic profile analyses, performed by ^1^H NMR-based metabolomic approach, indicated plant metabolites variations connected to the disease progression such as mannitol, quinic acid, and oleuropein related compounds. The best results, in terms of discrimination of the metabolic profiles with respect to water injection, were found for monthly endo-therapy treatments. Dentamet^®^ foliar application demonstrated more specific time related progressive effectiveness with respect to intravascular treatments. Therefore, besides a possible more effective performance of endo-therapy with respect to foliar treatments, the need of further doses/frequencies trimming to obtain long-term results was also assessed. The present field studies confirmed the indication of Dentamet^®^ effectiveness in metabolic variation induction, potentially linked with reducing the *X. fastidiosa* subspecies *pauca* related Olive Quick Decline Syndrome (OQDS) symptoms development.

## 1. Introduction

In Apulia, South–East Italy, over the last decade, the unexpected breakout of “Olive Quick Decline Syndrome” (OQDS) caused a significant agricultural and heritage loss, strongly affecting the local farmers economically [1]. As a result, olive groves are likely to become empty land in the Apulia peninsula, leading to a disruption of the ecosystem [2]. The first case of OQDS was identified by researchers in 2013 and from that day the disease is spreading not only to Salento’s southern parts but also into some northern areas, regardless of the preventive measures taken [3,4]. The bacterium *Xylella fastidiosa*, belonging to the *Xanthomonadaceae*, was retained as the pathogen responsible for the disease [5]. Taxonomically the known prominent subspecies of *X. fastidiosa* are *fastidiosa*, *multiplex*, and *pauca* [6,7,8]. Among these, the subspecies *pauca* (XFP) appears to be lethal to olive trees [9,10]. In the Mediterranean basin, the epidemic threat has not only affected the possible economic outcomes but also several cultivars possessing specific matchless traits. The OQDS is a severe disease, and it has spread through Lecce province and many hectares of the provinces of Brindisi, Taranto and areas north of Bari [11,12,13].

In the initial stage of the OQDS disease progression, the olive trees show leaf scorch and wilting of the branches that later overcome the whole tree canopy. Shriveled olive fruit and wrinkled leaves may remain attached to the desiccated shoots. More prominently, the twigs and trunk of the suffering tree show discoloration of xylem vessels as much as the XFP colonies prevail [14,15,16].

The European quarantine authorities have enlisted the XFP on the A2 list (n. 166) and the Regional Council of Apulia is working on an emergency plan. Nevertheless, still there is not a single complete cure that could fully eradicate the disease [17]. Generally, the policy to control bacterial disease is by preventing disease exposure by limiting contact with diseased plant material. In a situation when the bacterium has already spread out, the IMG (integrated management of the disease) by cultivating the resistant plants and controlling the disease vector can be helpful to overcome the disease. At the emergence of the OQDS disease spread-out, the only possible options were to eradicate and pruning of trees with clear symptoms. In the last few years, some chemical treatments proved to be useful in the partial treatment of the disease [18,19]. With some specific exceptions [20,21,22,23], the treatment of plant diseases by direct antibiotics application is globally forbidden due to several countable risks [24]. Therefore, due to the existing limitation, the potential solutions to treat the OQDS remain very few.

Early scientific reports suggested that copper has bactericidal action with the lowest risk of resistance development [25]. On the other hand, the use of common metal ions such as copper or zinc derivatives is sufficiently remarkable to disrupt the homeostasis of disease suffering plants, and the disturbance in metal ions balance can break-free the plant from the disease trap. Zinc and copper can be upheld in the xylem tract to interfere with the exopolysaccharide production and biofilm expansion of the *X. fastidiosa* in the vascular bundle [26,27]. Generally, the ionized compounds can disturb the biofilm of *X. fastidiosa* in the vascular bundle.

The research performed to optimize the bactericidal concentration of the ions showed that concentration of copper (>200 μM) and zinc (0.25 mM) inhibited biofilm formation in *X. fastidiosa* [28]. Another study suggests that planktonic cells are more vulnerable to copper concentrations, comparatively to biofilm [29]. Moreover, zinc can interfere with the biofilm exopolysaccharide formation [26,30], indicating that *X. fastidiosa* may need zinc detoxification prior to fully enable its virulence.

Some commercially available products such as Dentamet^®^ and NuovOlivo^®^ proved to be effectively useful against the OQDS disease in the plants affected by XFP [15]. The former consists of a copper, zinc and citric acid complex and was tested by our research group on the olive trees naturally suffering from Olive Quick Decline Syndrome associated with XFP. The commercially available, Dentamet^®^ is a mixture of copper (2% *w*/*w*) and zinc (4% *w*/*w*) complexed with fermentation derived citric acid hydracids [18]. Dentamet^®^ can be delivered to vascular tissue via foliar spray or trunk injection. Considering early results describing metal ions’ effectiveness on the pathogens, in most of our previous experiments, Dentamet^®^ was applied by foliar spray using an atomizer for the nebulization on the olive canopy. Positive results were obtained with foliar treatment as demonstrated by short and long-term experiments followed by NMR based metabolomics approach [31]. Being XFP a xylem-invading bacterium, the agro-active compound needs to be in contact with the pathogen in the xylem tissue. Therefore, topical spraying treatment may not be satisfactory in addition to being prohibitive in public areas. On the other hand, the goal of directly targeting the xylem vessels could be accomplished with trunk injection. Recently endo-therapy tests with systemic injections demonstrated efficiency for therapeutic purposes against the OQDS disease in XFP infected plants [32]. As expected, in the case of xylem limited pathogens, endo-therapy may increase the treatment efficiency by directly targeting the bacteria. Moreover, trunk injection/endo-therapy ensures the accurate translocation of the agro-active compound also in trees with a heavy infection, where due to a shortage of residual leaves, foliar treatment may be not effective. On the other hand, endo-therapy has several other benefits such as diminishing the chances of accidental dissipation of the agro-active compound into the environment and protecting the ecosystem in the long run [33]. Contrary to conventional chemical application methods, endo-therapy also limits the risk to farm workers.

We used a system called TIPS™, created by Invaio Sciences, that injects the active ingredients into the plant’s vessels with precision and speed. This system allows us to perform endo-therapy treatments in trees effectively. Invaio Sciences’ TIPS™ uses a 12 mm long, 1.5 mm thick, arrowhead-shaped injection tip to minimize plant damage and precisely deliver treatment formulations into trees’ active vasculature. The injection targets the vascular system, allowing superior uptake and distribution of treatment compounds at low pressure, between 1 and 3 bars. TIPS™ can handle a higher volume of injections than traditional systems and has a non-clogging design for efficient delivery [32,34].

Trees suffering heavily from OQDS in the Salento region were treated with Dentamet^®^ in a field experiment by endo-therapy or foliar spray and metabolic changes were quantified after 60, 120 and 180 days, from February to August 2021. In each treatment, all trees received over the focused timespan a mixture of water and Dentament^®^, except the control class that was endo-therapy treated with just water. Midterm metabolomic changes in these time points after treatments were analyzed by Nuclear Magnetic Resonance spectroscopy on leaf extracts. Previous NMR based endo-therapy related study showed in the leaf’s extracts, short-term changes in biomarkers compounds soon after the injection [32]. In the current study, a non-targeted ^1^H NMR fingerprinting was used and applied unsupervised and supervised pattern recognition techniques to observe how the olive trees that were naturally infected responded to the Dentamet^®^ treatments over a medium-term period [31,32,34,35], this work enables long-term monitoring of metabolic pathways modulation in infected olive trees after precision intravascular delivery of a Cu/Zn citric acid biocomplex (Dentamet^®^).

## 2. Results

### 2.1. Preliminary Unsupervised PCA Analysis: Whole Dataset

**Figure 1:**
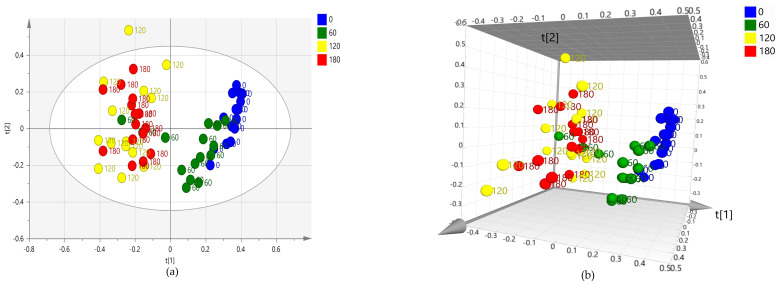
PCA (four components give R2X = 0.783, Q2 = 0.68) (**a**) t[1]/t[2] scores plot and (**b**) 3D scores plot for whole leaf samples dataset. Sample symbols are colored and labeled according to different days of the treatment: day 0 (before injection) and 60, 120 and 180 days after injection.

The unsupervised PCA analysis performed on the whole data set acquired by NMR showed the natural clustering of the samples from leaf extracts. A clear separation was showed upon the visual inspection of the scores plot, along the first component t[1] of time 0 and time 60 samples, with respect to time 120 and 180 (days from the first treatment) (Figure 1a,b). Moreover, a discrimination of the metabolic profiles of time 0 and time 60 samples could be observed along the second component t[2], indicating a substantial effect on the samples related to the seasonal progression.

**Figure 2:**
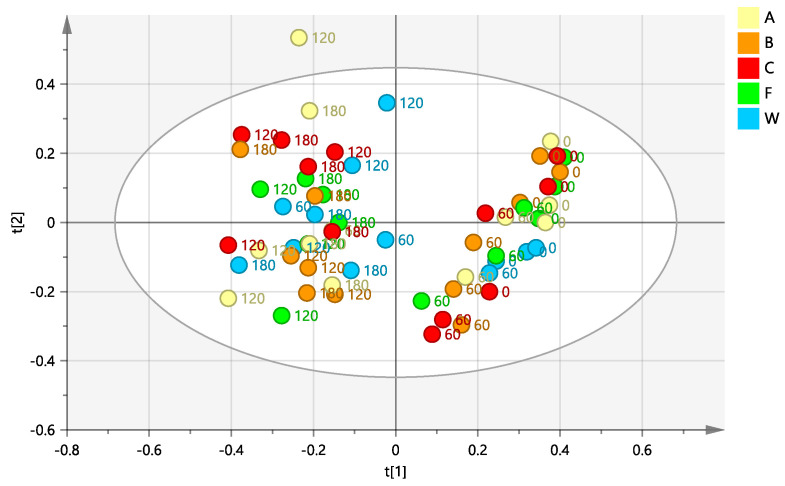
PCA scores plot (four components give R2X = 0.783, Q2 = 0.68) for whole leaf samples dataset, Sample symbols are colored according to different treatment types: A: low dose monthly treated samples (by trunk injection) B: high dose monthly treated samples (by trunk injection), C: high dose bimonthly treated samples (by trunk injection); W: monthly water injected samples (by trunk injection); F: foliar sprayed monthly treated samples and labeled according to different days of the treatment: day 0 (before injection) and 60, 120 and 180 days after injection.

Interestingly, as better seen in the 3D scores plot Figure 1b, three of time 60 samples were observed in the time 120/180 region, indicating for them a metabolic profile similar to that exhibited after prolonged treatment (120/180 days). Labeling the PCA scores plot of Figure 1b according to the treatment performed revealed that the three samples belong to two water and one low dose-treated (Figure 2). This may suggest an effect of the treatments in slowing down the metabolic changes normally occurring in water-treated samples according to the season progression.

### 2.2. Supervised PLS-DA Analysis: Time Sample Classes (0, 60, 120, 180 Days)

**Figure 3:**
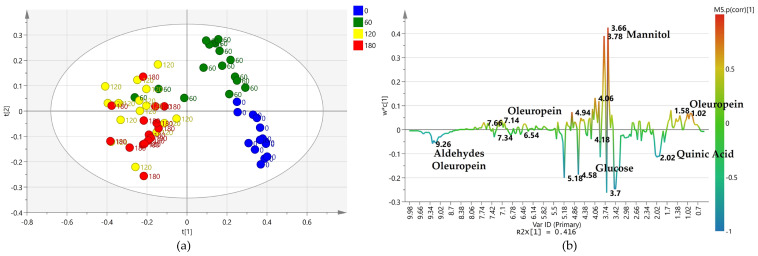
(**a**) PLS-DA scores plot (four components give R2X = 0.709, R2Y = 0.689, Q2 = 0.566) for leaf samples from different days of the treatment: day 0 (before injection) and 60, 120 and 180 days after injection (**b**) S line plot for the model colored according to the correlation-scaled coefficient (p(corr)).

A supervised PLS-DA analysis was performed to deeply investigate the natural grouping of the samples (observed by unsupervised PCA), considering the time sample classes. The differentiation of the sample classes, defined according to the sampling time, confirmed the already described separation, observed in the preliminary PCA plot. In particular, along the first component t[1] a clear distinction was observed of time 0 and time 60 sample classes, with respect to time 120 and 180 (days from the first treatment) (Figure 3a). Moreover, as already observed in the PCA plot, a much clearer differentiation (along t[2]) could be found between time 0 and time 60 with respect to time 120 and 180 classes.

The S-line plot for the model (Figure 3b) revealed the molecules responsible for the observed separation along the t[1] component. In particular, a higher relative content of oleuropein (at 1.09, 2.72, and 4.1 ppm) and mannitol (at 3.74 ppm) was observed for day 0 and day 60 with respect to day 120/180 sample classes.

### 2.3. Supervised Pairwise OPLS-DA Analysis: Time 0 vs. All the Treated Samples (Time 60, 120, and 180 Days)

**Figure 4:**
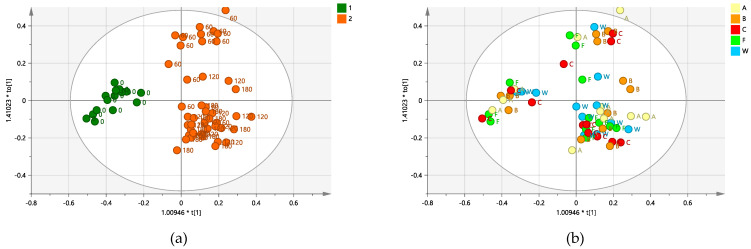

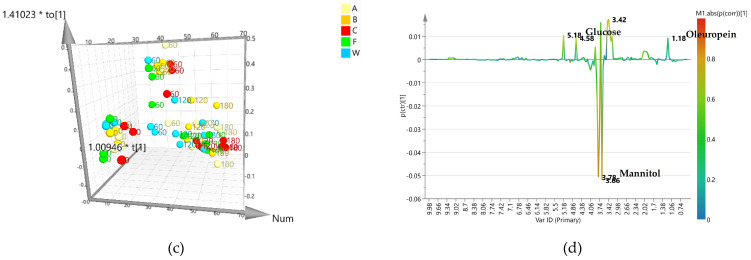
(**a**) OPLS-DA scores plot (1 + 1 + 0 components give R2X = 0.504, R2Y = 0.839, Q2 = 0.805) for time 0 and treated (time 60, 120, and 180) class leaf samples. (**b**) Scores plot for the model with samples symbols colored according to the treatment. (**c**) 3D scores plot for the model with samples symbols colored according to the treatment. (**d**) S line plot for the model colored according to the correlation-scaled coefficient (p(corr)). Sample symbols are labeled according to different days of the treatment: day 0 (before injection) and 60, 120 and 180 days after injection.

A further pairwise analysis (OPLS-DA) comparing time 0 with all the treated samples (time 60, 120, and 180) considered as a single class, shows a clear differentiation between the two set classes. Moreover, samples after day 60, 120, and 180 appeared distributed along the first orthogonal component to [1] (Figure 4a). Most of the day 60 scores appear strongly differentiated from most of the 120 and 180 days samples with a small mixed group (60, 120 and 180 days) separating along the first orthogonal component to [1] the two major clusters (60 and 120/180 days).

As already seen in the PCA and PLS-DA analyses (Figure 1 and Figure 3), the 120 and 180 days samples showed similar close grouping. In contrast, day 60 samples exhibited more significant differentiation along the first orthogonal component. On the other hand, no clear differentiation along the orthogonal component could be observed, analyzing the same OPLS-DA score-plot, when the samples are labeled according to the different treatment (Figure 4b). Nevertheless, as already discussed, two water injected and one low dose treated from day 60 samples show a close similarity to the days 120 and 180 sample groups as observed in the 3D scores plot (Figure 4c). Furthermore, the S-line plot revealed the higher relative content of mannitol in the water-injected samples compared to those treated with the biocomplex (Figure 4d).

In conclusion, examining data by both unsupervised (PCA) and supervised (PLS-DA and OPLS-DA) analyses, the primarily observed clustering and differences in the samples appeared to be essentially related to the time progression rather than the performed treatment. Interestingly, in the case of supervised analyses, these results were also buttressed by a four classes PLS-DA model (according to days from treatment) and further confirmed by OPLS-DA, comparing with time 0 all the treated samples (time 60, 120 and 180), considered as a single class.

### 2.4. Supervised OPLS-DA Analysis: Biocomplex Based versus Water Treatments

**Figure 5:**
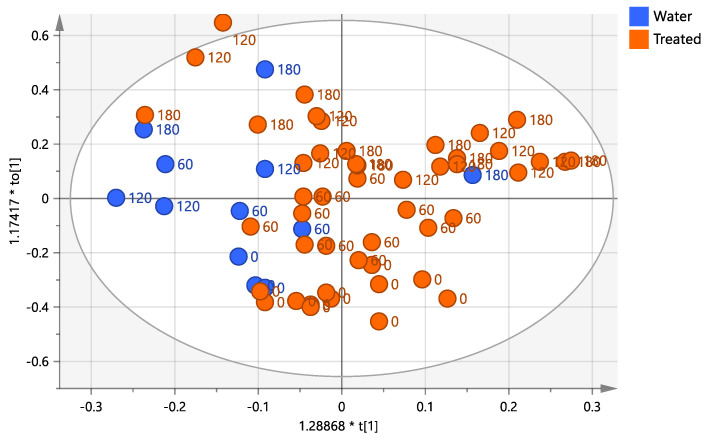
OPLS-DA scores plot (1 + 1 + 0 components give R2X = 0.463, R2Y = 0.225, Q2 = 0.0347) for all water and Dentamet^®^ treated class samples. Sample symbols are labeled according to the different days of the treatment: day 0 (before injection) and 60, 120 and 180 days after injection.

In order to focus on the treatments’ effects, a general comparison of all biocomplex treatments with respect to all water treated samples was carried out. The resulting OPLS-DA pairwise model was characterized by very low model quality parameters (Figure 5).

Clearer information was further provided by several specific OPLS-DA pairwise analyses. These involved specific comparisons between selective biocomplex treated samples, subject to a single specific bio-complex treatment and water-treated samples. The OPLS-DA pairwise models selectively focused also on each of the sampling times (0, 60, 120, and 180 days from the treatment).

#### 2.4.1. OPLS-DA Pairwise Analysis—Monthly Foliar versus Water Treatment

**Figure 6:**
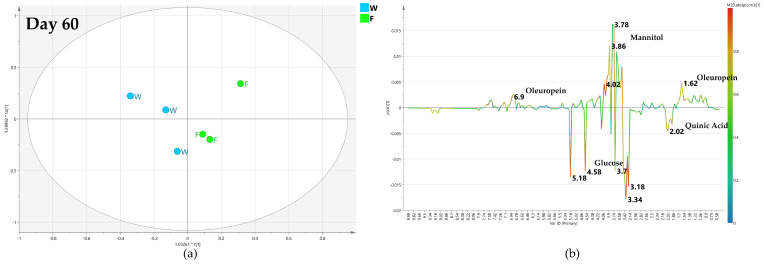
(**a**) OPLS-DA scores plot (1 + 1 + 0 components give R2X = 0.838, R2Y = 0.727, Q2 = 0.232) for day 60 foliar (F) and water (W) class leaf samples, (**b**) S line plot for the model colored according to the correlation-scaled coefficient (p(corr)).

After 60 days of monthly treatment, the OPLS-DA comparison between biocomplex foliar and water treatment gave a relatively poor predictive model with Q2 = 0.232 (Figure 6a). The two considered classes were quite close at the 0 value of the predictive component t[1]. Considering a warning for the poor model predictivity, the analysis of the relative S-line plot showed a higher relative content of mannitol (binned at 3.78 ppm) and oleuropein (binned at 1.62 and 6.9 ppm) in biocomplex foliar treated samples while water treated samples were characterized by relatively higher glucose (5.18–4.58 ppm) and quinic acid (2.02 ppm) content (Figure 6b).

**Figure 7:**
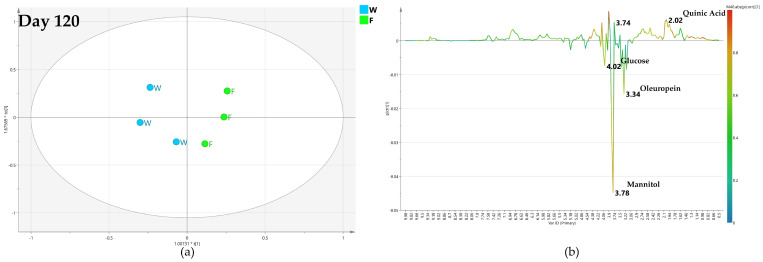
(**a**) OPLS-DA scores plot (1 + 1 + 0 components give R2X = 0.834, R2Y = 0.857, Q2 = 0.445) for day 120 foliar (F) and water (W) class leaf samples, (**b**) S line plot for the model colored according to the correlation-scaled coefficient p(corr).).

After 120 days, the quality of the OPLS-DA model, comparing the foliar biocomplex versus water treatment, slightly increases as described by the value of the predictive parameter (Q2 = 0.445) (Figure 7a). It was shown by the S line plot for the model that the water treated samples had a highly increased relative content of mannitol (3.78 ppm). Yet, a relatively higher quantity of quinic acid (2.02 ppm) was observed in the biocomplex foliar treated samples (Figure 7b).

**Figure 8:**
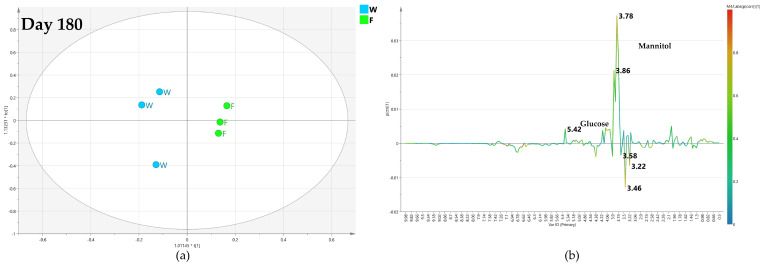
(**a**) OPLS-DA scores plot (1 + 1 + 0 components give R2X = 0.661, R2Y = 0.97, Q2 = 0.467, Q2 = 0.445) for day 120 foliar (F) and water (W) class leaf samples, (**b**) S line plot for the model colored according to the correlation-scaled coefficient p(corr).

After 180 days of monthly foliar treatment, the predictive ability of the OPLS-DA model still slightly increased (Q2 = 0.445) as the differences in the metabolic profiles of the two considered class samples are quite different (Figure 8a). As observed from the S line plot for the model, the relative content of mannitol (3.78 ppm) for biocomplex foliar-treated samples is higher than in water treated samples. These latter, on the contrary, exhibited a relatively higher content of oleuropein (1.18 ppm) (Figure 8b).

Among the biocomplex monthly foliar treated samples, the highest treatment impact was observed for samples collected after 180 days showing OPLS-DA with Q2 = 0.467. A mediate effect was recorded in the day 120 collected samples model with a Q2 = 0.445, and the lowest effect was observed in day 60 samples (Q2 = 0.232) (Figure 6, Figure 7 and Figure 8). The models obtained by pairwise analysis from foliar spray treatment samples show a gradual increase in treatment effect, suggesting that treatment by foliar sprinkle has a slow but long-term acting impact.

#### 2.4.2. OPLS-DA Pairwise Analysis—Monthly Low Dose Endo-Therapy versus Water Treatment

**Figure 9:**
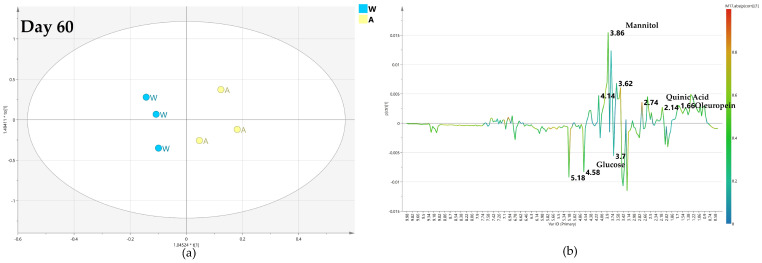
(**a**) OPLS-DA scores plot (1 + 1 + 0 components give R2X = 0.774, R2Y = 0.89, Q2= −0.57) for day 60 monthly low dose (A) and water (W) class leaf samples, (**b**) S line plot for the model colored according to the correlation-scaled coefficient p(corr).

A specific comparison was then performed between monthly low-dose biocomplex endo-therapy and water treated class samples. For the samples collected after 60 days, the obtained OPLS-DA model is characterized by poor predictivity with a negative value (−0.57) of the Q2 parameter (Figure 9a), although a separation between the two considered classes (water versus biocomplex endo-therapy-treated samples) could be observed. Thus, no specific information could be obtained from the relative S-line plot (Figure 9b).

**Figure 10:**
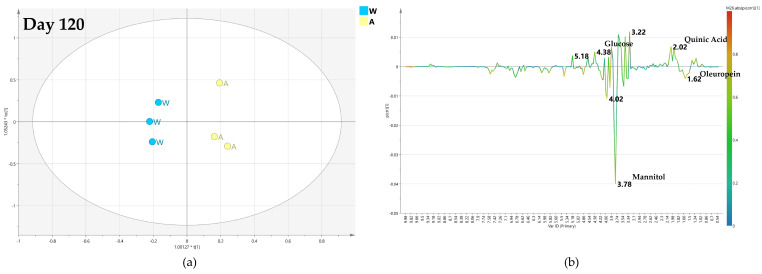
(**a**) OPLS-DA scores plot (1 + 1 + 0 components give R2X = 0.731, R2Y = 0.982, Q2 = 0.621) for day 120 monthly low dose (A) and water (W) class leaf samples, (**b**) S line plot for the model colored according to the correlation-scaled coefficient p(corr).

The predictive ability (Q2 = 0.621) of the OPLS-DA model is greatly enhanced in the comparison performed for the samples collected after 120 days of monthly low dose biocomplex vs. water endo-therapy (Figure 10a). The relative S line plot showed higher mannitol content (3.78 ppm) for the water with respect to biocomplex treated samples (Figure 10b). In turn, this latter showed a relatively higher content of oleuropein (1.18 ppm), quinic acid (2.02 ppm) and glucose (5.18, 4.58 ppm) (Figure 10b).

**Figure 11:**
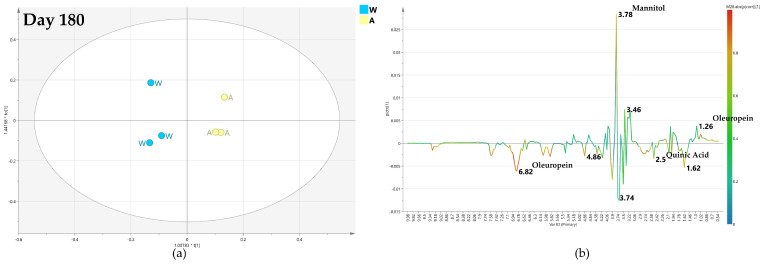
(**a**) OPLS-DA scores plot (1 + 1 + 0 components give R2X = 0.222, R2Y = 0.982, Q2 = 0.103) for day 180 monthly low dose (A) and water (W) class leaf samples, (**b**) S line plot for the model colored according to the correlation-scaled coefficient p(corr).

Interestingly, at day 180, the resulting OPLS-DA was described by poor model predictivity parameter (Q2 = 0.103) (Figure 11a). Nevertheless, some limited indications were obtained for higher mannitol (at 3.78 ppm and 3.86) in low dose treated samples and relatively higher content of oleuropein (at 1.18 and 6.82 ppm) in water-injected samples (Figure 11b).

For the models comparing monthly biocomplex low-dose endo-therapy and water-treated samples, the maximum class discrimination was attained after 120 days of treatment. Much lower discriminations were obtained at shorter (day 60) or longer (day 180) treatment periods (Figure 9, Figure 10 and Figure 11). This behavior contrasted sharply with the observed results in the pairwise OPLS-DA models of monthly biocomplex foliar vs. water treatment.

#### 2.4.3. OPLS-DA Pairwise Analysis—Monthly High Dose Endo-Therapy versus Water Treatment

**Figure 12:**
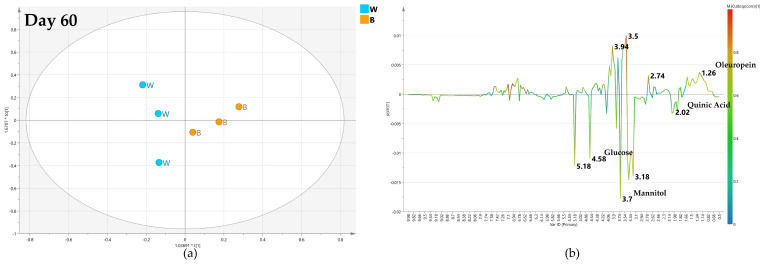
(**a**) OPLS-DA scores plot (1 + 1 + 0 components give R2X = 0.738, R2Y = 0.831, Q2 = −0.053) for day 60 monthly high dose (B) and water (W) class leaf samples, (**b**) S line plot for the model colored according to the correlation-scaled coefficient p(corr).

In the comparison of monthly high dose biocomplex versus water treatment, it could be observed as, after 60 days, the resulting model was poorly predictive with a negative value (−0.053) of the Q2 parameter, suggesting a descriptive although not sound discrimination between the class samples. Thus, no specific information could be obtained from the relative S-line plot (Figure 12b).

**Figure 13:**
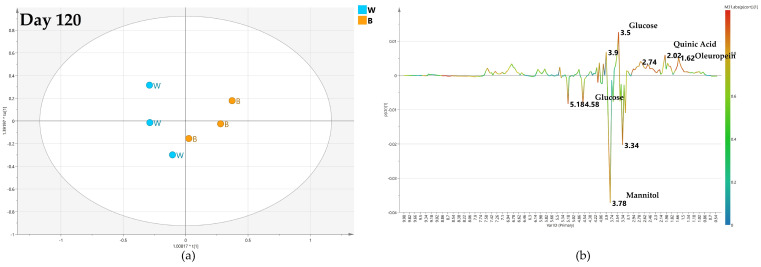
(**a**) OPLS-DA scores plot (1 + 1 + 0 components give R2X = 0.848, R2Y = 0.781, Q2 = 0.551) for day 120 monthly high dose (B) and water (W) class leaf samples, (**b**) S line plot for the model colored according to the correlation-scaled coefficient p(corr).

Interestingly, after 120 days, the relative OPLS-DA model for comparison with water treatment was described by a good predictive parameter (Q2 = 0.551). The S-line plot for the model revealed a higher relative content of mannitol (binned at 3.78 ppm) and α/β glucose (at 4.58 and 5.18 ppm) for water-injected samples. In comparison, quinic acid (at 2.02 ppm) and oleuropein (at 1.62 ppm) were detected in a relatively higher amount in monthly high-dose treated samples (Figure 13).

**Figure 14:**
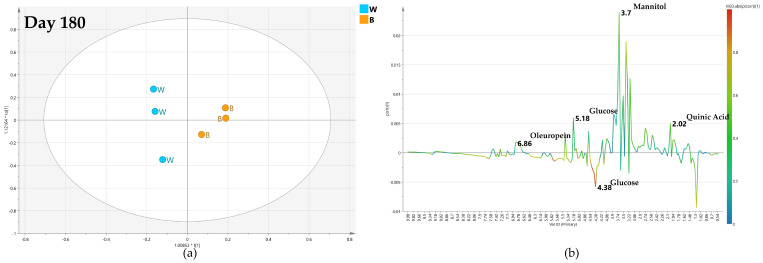
(**a**) OPLS-DA scores plot (1 + 1 + 0 components give R2X = 0.514, R2Y = 0.926, Q2 = 0.47) for day 180 monthly high dose (B) and water (W) class leaf samples, (**b**) S line plot for the model colored according to the correlation-scaled coefficient p(corr).

After 180 days from the treatment, despite a slight decrease of the predictive parameter (Q2 = 0.47), the OPLS-DA model, comparing monthly high-dose biocomplex endo-therapy versus water samples, was still found well differentiating the classes (Figure 14a) In this case, the S-line plot showed higher content of oleuropein (1.18 ppm) in water-treated samples. Meanwhile, higher content of mannitol (at 3.7 ppm) and glucose (at 5.18 and 5.42 ppm) showed in high-dose biocomplex treated samples, revealed an inversion of the trend observed in the day 120 model (Figure 14b).

According to the described OPLS-DA models, the monthly high-dose biocomplex endo-therapy exhibited some promising effects with respect to monthly low-dose treatment. In both cases, the model predictivity index resulted negative after 60 days (Q2 = −0.57 and −0.053 for the models of Figure 9a and Figure 12a, respectively). A strong improvement in class discrimination was also observed for both low and high doses of biocomplex endo-therapy vs. water after 120 days (Q2 = 0.621 and 0.551 for the models of Figure 10 and Figure 13, respectively). Nevertheless, the biocomplex vs. water class discrimination was sustained after 180 days for the monthly high dose treatment (Q2 = 0.103 and 0.47 for the models of Figure 11 and Figure 14, respectively). Accordingly, the endo-therapy treatment impact with a monthly high-dose biocomplex seemed to show better endurance (Figure 12, Figure 13 and Figure 14), possibly helping the plant to sustain the infection in the long run and overcome the seasonal disease progression.

#### 2.4.4. OPLS-DA Pairwise Analysis—Bimonthly High Dose Endo-Therapy versus Water Treatment

**Figure 15:**
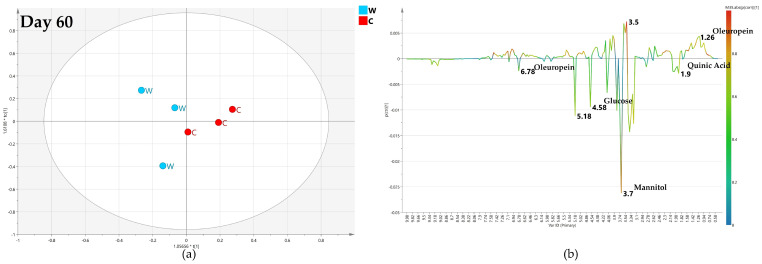
(**a**) OPLS-DA scores plot (1 + 1 + 0 components give R2X = 0.753, R2Y = 0.726, Q2 = −0.45) for day 60 bimonthly high dose (C) and water (W) class leaf samples, (**b**) S line plot for the model colored according to the correlation-scaled coefficient p(corr).

Finally, the specific bimonthly high-dose biocomplex endo-therapy vs. water treatment comparison was analyzed. As assessed by the negative value (Q2 = −0.45) for the predictive model parameter, no specific separation between the two classes could be observed after 60 days (Figure 15a). Thus, no specific information could be obtained from the relative S-line plot (Figure 15b).

**Figure 16:**
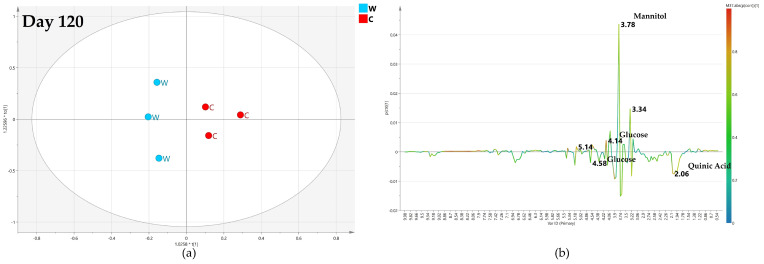
(**a**) OPLS-DA scores plot (1 + 1 + 0 components give R2X = 0.726, R2Y = 0.881, Q2 = 0.366) for day 120 bimonthly high dose (C) and water (W) class leaf samples, (**b**) S line plot for the model colored according to the correlation-scaled coefficient p(corr).

The quality of the OPLS-DA model slightly improved in the day 120 treatment comparison (Figure 16a). The S-line plot reveals the different metabolic profiles for the two considered classes. Specifically, a higher relative content of mannitol (binned at 3.78 ppm) was detected in bimonthly high dose biocomplex endo-therapy treated samples, while higher quinic acid (2.02 ppm) was observed in water-injected samples (Figure 16b).

**Figure 17:**
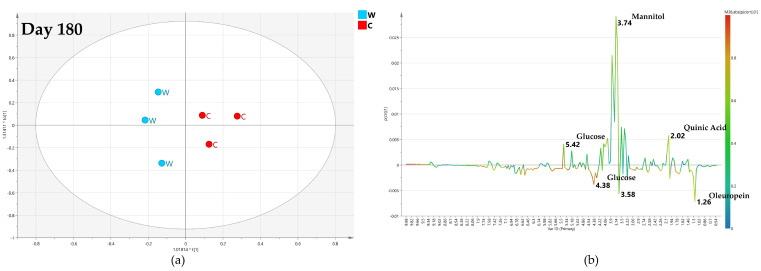
(**a**) OPLS-DA scores plot (1 + 1 + 0 components give R2X = 0.644, R2Y = 0.87, Q2 = 0.16) for day 120 bimonthly high dose (C) and water (W) class leaf samples, (**b**) S line plot for the model colored according to the correlation-scaled coefficient p(corr).

After 180 days, the discrimination of the two classes returned to be very low (Q2 = 0.16), suggesting a possible decline in the long-term effect for the bi-monthly high-dose biocomplex endo-therapy-treated samples (Figure 17a). Thus, no specific information could be obtained from the relative S-line plot (Figure 17b).

### 2.5. Pairwise OPLS-DA Analyses Biocomplex vs. Water Treatments: Predictive Model Parameters Overview

**Figure 18:**
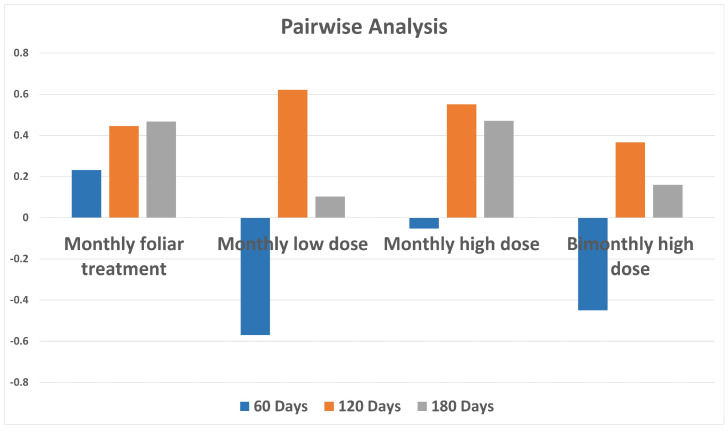
Bar chart of Q2 statistical parameter values of OPLS-DA models for biocomplex vs. water treatment leaf samples. Q2 is a goodness of prediction parameter representing the portion of the variance in the data predictable by the model. The negative Q2 score at day 60 in all three endotherapy treatments is an indication of low predictivity.

Some interesting points could be obtained by comparing the predictive model parameters (Q2) for all pairwise OPLS-DA analyses. These include foliar and all doses of endo-therapy biocomplex treatments with respect to water injection, considered for all the focused sampling periods. A graphical summary of all the predictive parameter values (Q2) for the models (Figure 6, Figure 7, Figure 8, Figure 9, Figure 10, Figure 11, Figure 12, Figure 13, Figure 14, Figure 15, Figure 16 and Figure 17), obtained by comparing with water-injected samples, one by one monthly foliar sprinkle, low-dose treated, high dose-treated and bi-monthly high-dose treated samples, each time, is reported in Figure 18. The discrimination, with respect to water injection, in the result of foliar treatments seemed to be increasing continuously from day 60 to 120 and 180, which hints that foliar spray takes time to effectively be absorbed by plants to express the complete results. On the other hand, under the used conditions, within the first 60 days, the endo-therapy did not result in a discrimination effect improvement with respect to the foliar treatment. Nevertheless, it appears that 120 days of monthly endo-therapy treatments with low or high doses resulted more effective than foliar spray in producing a clear discrimination with respect to water treatment.

Comparing the bi-monthly high dose versus water with the monthly high dose versus water OPLS-DA models, further interesting results could be obtained. The class discrimination trend, considering the treatment progression after 60, 120 and 180 days, appeared similar in both cases with an observed minimal Q2 value in the first model (after 60 days) which increases (after 120 days) to show further a relatively small reduction (after 180 days). Nevertheless, the Q2 values related to monthly high dose models (Q2 = −0.053, 0.551, and 0.47 for Figure 12, Figure 13 and Figure 14, respectively) are notably higher with respect to corresponding bi-monthly high dose (Q2 = −0.45, 0.366 and 0.16 for Figure 15, Figure 16 and Figure 17 respectively). On the other hand, a comparison of bi-monthly high dose vs. water with the low monthly dose vs. water OPLS-DA models only showed a substantial beneficial effect in class separation after 120 days of treatment for low monthly dose (Q2 = −0.57, 0.621, and 0.103 for Figure 9, Figure 10 and Figure 11, respectively) with respect to (Q2 = −0.45, 0.366 and 0.16 for Figure 15, Figure 16 and Figure 17, respectively).

After 180 days, the monthly endo-therapy seemed to be generally less effective than after 120 days in discrimination with respect to water treatment, but this could also be a result of specific seasonal disease progression. Moreover, the effect of a high-dose, after 180 days of endo-therapy, was still comparatively more discriminating vs. water injection, with respect to foliar treatment. Finally, it should be noted that the high dose bimonthly appeared to be generally less effective, on average, than the analogous monthly treatment, in producing discrimination with respect to water, as clearly shown by the specific models’ predictive parameters.

### 2.6. Pairwise OPLS-DA Analyses Biocomplex vs. Water Treatments: Fold Change Analysis of Discriminating Metabolites

The multivariate analysis based on Nuclear Magnetic Resonance spectra assessed the significant variations in the discriminant metabolite content. The quantitative comparison between biocomplex and water treated leaf samples for discriminating metabolites was then performed by considering the fold change (FC) ratio.

**Figure 19:**
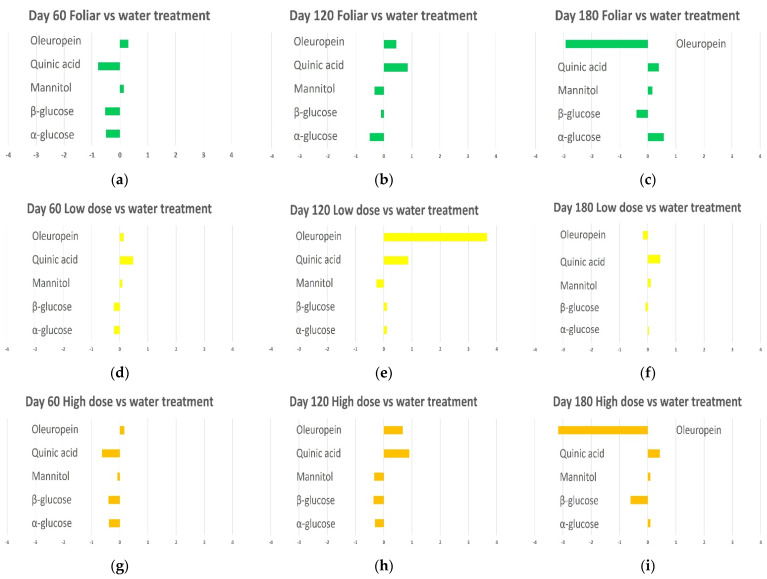

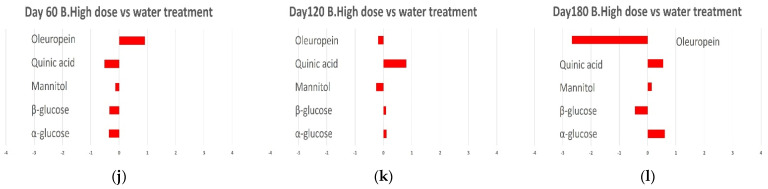
Graphical summary of discriminant metabolite comparison among biocomplex vs. water treated leaf samples. The *X*-axis reports log2 fold change (FC) values. (**a**) Day 60 monthly foliar vs. water treatment, (**b**) Day 120 monthly foliar vs. water treatment (**c**) Day 180 monthly foliar vs. water treatment (**d**) Day 60 monthly low dose vs. water treatment (**e**) Day 120 monthly low dose vs. water treatment (**f**) Day 180 monthly low dose vs. water treatment (**g**) Day 60 monthly high dose vs. water treatment (**h**) Day 120 monthly high dose vs. water treatment (**i**) Day 180 monthly high dose vs. water treatment (**j**) Day 60 bimonthly high dose vs. water treatment (**k**) Day 120 bimonthly high dose vs. water treatment (**l**) Day 180 bimonthly high dose vs. water treatment.

Therefore, fold changes (FCs) were calculated from the buckets’ integral values related to the relevant NMR signals for oleuropein, quinic acid, mannitol, α and β glucose (Figure 19). Interestingly, fold change profiles discriminating with respect to water appear very similar, for all the considered metabolites, in high dose and foliar treatments. This demonstrates that the closest behavior indicated by the model predictive parameter (Q2) observed for high-dose and foliar in the discrimination with respect to water treatment (Figure 18) was also accompanied by the same kind of metabolite variation. A similar type of metabolites’ variation with respect to water was also observed when comparing bimonthly high dose and foliar treatment.

On the other hand, some slight mismatching variation could also be observed in this case, especially for the 120 days samples (particularly oleuropein variation). Interestingly the most different metabolites variation profiles, with respect to water, were observed comparing the low dose with foliar treatment. Nevertheless, the 120 days samples resulting from both low-dose and foliar treatments showed a similar fold change profile when considering oleuropein, quinic acid, and mannitol. It should also be noted that the low dose treatment after 120 days resulted in the best Q2 parameter when comparing the pairwise OPLS-DA models obtained for biocomplex treatments with respect to water (Figure 18). According to the fold changes profiles depicted in Figure 19, the much higher increase of oleuropein observed when comparing low dose vs. foliar treatment results, with respect to water, may be responsible for this outcome.

**Figure 20:**
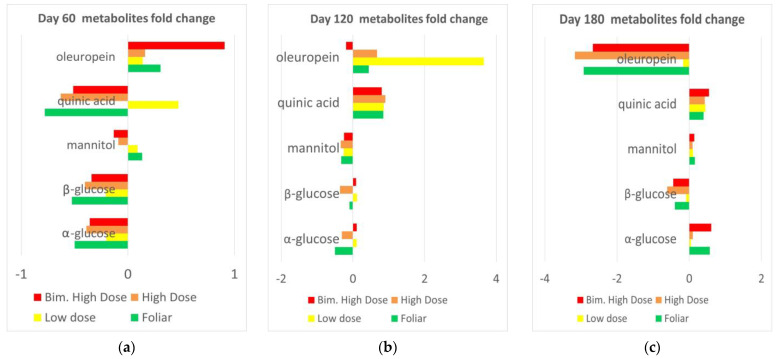
Comparative graphical summary of discriminant metabolite comparison among biocomplex vs. water treated leaf samples. The *X*-axis reports log2 fold change (FC) values. (**a**) Day 60 biocomplex vs. water treatment, (**b**) Day 120 biocomplex vs. water treatment (**c**) Day 180 biocomplex vs. water treatment.

Observed trends of fold change for the selected metabolites according to the progression of time, for the different performed treatments (with respect to the water control) could be summarized in Figure 20. The initial lower oleuropein content observed in the biocomplex with respect to the water treated samples appeared essentially reverted from day 60 to day 120 (except in bimonthly high dose treatment) and completely reverted at day 180. A nearly opposite trend follows the quinic acid initially lower in the biocomplex with respect to the water-treated samples (at day 60, except in low dose treatment) and therefore found to be higher than control in both day 120 and 180 samples for all the treatments. Interestingly, the mannitol, initially (day 60) slightly higher (foliar and low dose) and lower (monthly and bimonthly high dose) in comparison to the water treated samples, became generally slightly lower (day 120) or higher (day 180) in comparison to controls in further sampling. A general smoothing of the differences observed for the other sugars (α and β glucose) with respect to controls was also observed with the sampling time progression. The initially marked lower content (α and β glucose) in biocomplex treated, with respect to water treated samples, appear much less evident and, in some cases, reverted when considering day 120 and 180 observations. The list of the parameters and the figures for all the analyses was reported in Supplementary Material as Table S2.

## 3. Discussion

The epidemic of OQDS in Apulia due to XFP is challenging for researchers. There are several ongoing investigations, to possibly achieve plant pathology management [36,37,38]. In the present study, the effects of foliar and trunk injection of Cu/Zn biocomplex were assessed by ^1^H-NMR and MVA, along the different seasons, at different dosages and treatment times in the metabolic profiles of leaf extracts in XFP infected trees. The endo-therapy was performed with a novel proprietary precision injection system (TIPS™, designed by Invaio Sciences, Cambridge, USA). A total of 60 leaf extracts, from 15 naturally infected trees were analyzed to observe the effect of different applications of Dentamet^®^ (foliar and endo-therapy) after 60, 120 and 180 days. Five treatment categories were performed and then analyzed: monthly endo-therapy with low dose (A), monthly endo-therapy with high dose (B), bimonthly endo-therapy with high dose (C), monthly foliar application (F), and monthly injection with water (W) as a control.

The studied plants were located in the well-established disease infected area of Apulia (Salento), moreover, the presence of XFP bacteria was confirmed through the use of quantitative real-time PCR (qPCR). The metabolic profile analyses, performed by ^1^H NMR-based MVA metabolomic approach, reveal the fluctuations in the plant’s metabolites connected to the disease progression, such as mannitol, quinic acid and oleuropein-related compounds. The preliminary unsupervised and supervised analyses reveal a clear separation of time 0 and time 60 (treatment days) samples with respect to time 120, and time 180. This suggests that, besides the effects of the performed treatments, a major contribution to the sample differences is related to seasonal progression.

Further supervised multivariate analyses provided some indications of the specific treatment impact on the plants. Clearer information was obtained from OPLS-DA pairwise models, comparing, for each sampling time, selective biocomplex applications with respect to simple water treatments. In this respect, the resulting predictivity values (Q2) clearly indicate the observed discrimination degree for the specific treatment with respect to water-treated controls. Foliar application of the biocomplex (Dentamet^®^) was noticed for its specific time-related progressive discrimination with respect to water treated controls when compared to intravascular treatments. Nevertheless, the best results, in terms of discrimination with respect to water injection, were found for endo-therapy treatments. Indeed, at day 120, monthly endo-therapy with low or high dose biocomplex treatment shows vs. water better discrimination of metabolic profiles than foliar treatments.

Interestingly, after 180 days, the monthly endo-therapy seems to be generally less effective than after 120 days, although this may be a result of specific seasonal disease progression. Finally, a noticeable distinction was found comparing high dose to low dose Dentament^®^ treated plants by endo-therapy. Remarkably, the effect after 180 days for monthly high-dose, compared to low dose treated samples, resulted in a longer lasting higher discrimination, with respect to water treated controls. On the other hand, with respect to monthly, the bi-monthly endo-therapeutic high dose treatment showed lower discrimination effects versus water controls. It should also be noted that the above-discussed discriminations levels, observed in the OPLS-DA pairwise models, stem from metabolites variation occurring in the specific biocomplex with respect to water-treated control samples. These metabolite variations have been thoroughly analyzed as specific observed fold changes in the treated with respect to control samples.

The identified discriminant metabolites of treated and control samples have been compared considering the FC ratios. One of the most abundant and defensive compounds that help olive plants cope with drought stress is the secoiridoid oleuropein [39]. The general time decreasing trend for oleuropein related compounds content, observed in all treated samples, suggests a possible straightforward stimulation of the phenylpropanoid pathway by biocomplex treatment, as we already described [32]. Thus, the absence of a specific treatment could describe the much slower and less significant response by untreated controls that leads to an increase in the oleuropein content only at day 180, as a physiological response to the disease. Moreover, together with oleuropein, also mannitol is known to participate in protection against drought stress in olive plants [40]. The observed complex trend, according to which, mannitol content is initially slightly higher (low dose and foliar treatment) or lower (high dose and bimonthly high dose) for specific treatments, with respect to the water treated samples and became slightly lower (day 120) or higher (day 180) with respect to controls in further sampling could be possibly explained by different considerations. The observed higher content of the sugar (mannitol) in foliar treated samples at day 60 is in accordance with the already reported results [35,39], related to improved physiological performance and photosynthetic capacity of olive trees. However, the mannitol content clearly decreased as a specific consequence of endo-therapy from day 60 to day 120 for all the treatments, which is consistent with our previous results that showed a similar trend [32]. Nevertheless, a seasonal effect of the mannitol content pattern that peaks in summer [41] should be taken into account by observing the clear increase of its content at day 180 (August sampling). Like mannitol, a lower content of glucose observed in treated samples with respect to controls suggests a specific response to the biochemical treatments, according to our previous work [32]. In this context, the higher content of the mannitol in control samples, observed at day 60, could be seen because of a cascade of defense responses against disease to increase carbon uptake, as described in previous work [32]. The specific trend observed for the quinic acid, according to which biocomplex treated samples (except in low dose treatment) exhibited, at day 60, a lower content with respect to water controls, confirms the already reported [31,32], decrease of this disease biomarker compound as a specific effect of the treatment. Moreover, the specific pattern of quinic acid concentrations, which depend on the temperature and reach the highest level during the hottest period of the year for other plants, could explain the increase in both, day 120 and 180 samples for all the treatments [42]. As reported in previous studies, the season shapes the biochemical makeup of olive leaves, creating diverse and dynamic changes in their quality and quantity. In addition, other factors such as the plant cultivar, climatic conditions, sampling period, genetics, and geographical origin should be considered [43]. Therefore, a specific assessment of the seasonal effects on the chemical composition of olive leaf extracts is essential to ensuring optimal treatment doses/frequencies to obtain long-term results. Similar importance is expected for the monitoring of the biomarker compounds also during the winter period when no treatment is released to the tree and the metabolic activity of the plant is markedly different from the one analyzed in the present study. The obtained results show that repetitive trunk injection with the biocomplex results in a metabolic profile change with respect to water treatment, which can correlate to an overall crop health improvement and possibly to (partial) pathogen activity control. Comparisons with foliar treatments show a possible achievement of higher metabolic plant response, although further specific refinements and optimizations related to dose, and treatment frequency are required.

On the other hand, too high concentrations of Cu/Zn complex also (potentially) raise the secondary problem of phytotoxicity. The literature suggests that the excessive dosage of copper and zinc with the injection may cause oxidative stress because it creates an imbalance between antioxidant responses and increases the production of reactive oxygen species (ROS) [44,45]. The available data interlink the high level of Cu in plants with a disturbance of bio-regulatory processes, such as inference in chromatin structure, and obstruction of enzymatic activities involved in photosynthesis and respiration [46]. Meanwhile, a high level of Zn lowers the antioxidant defense system of plants by producing oxidative stress, and it also downregulates photosynthetic efficiency [47,48]. Therefore, although promising, Cu/Zn biocomplex based endotherapy clearly requires further specific trimming related to dose, and treatment frequency for an optimal outcome.

## 4. Methods

### 4.1. Trunk Injection and Foliar Spray Treatments of Infected Olive Trees

Starting from February 2021, fifteen trees with an average age of 150-year-old, olive cv. Ogliarola Salentina and Cellina di Nardo located in Uggiano La Chiesa (Lecce province, Salento peninsula) were selected that were severely suffering from OQDS by XFP. The trees were selected for testing the prospect of recovery from the disease through the foliar spray and trunk injection of Dentamet^®^ (Diagro, Bergamo, Italy). The experiment was designed into five categories, based on the mode/dose of plant treatment: monthly endo-therapy with low dose treated with 25 mL (Dentamet^®^) + 50 mL (water), monthly endo-therapy with high dose treated with 50 mL (Dentamet^®^) + 100 mL (water), bimonthly endo-therapy with high dose treated with 50 mL (Dentamet^®^) + 100 mL (water), monthly foliar application treated with 50 mL (Dentamet^®^) + 14.95 L (water), and monthly injection with just water treated with 75 mL (water). Intravascular treatment was performed using the Invaio Sciences (Cambridge, MA, USA) proprietary TIPS^TM^ microinjection system [32,34]. A single injection point was set at the base of the trunk of each tree. A disposable pressurized canister (1 to 3 bar) was filled with different rates of Dentamet^®^. A small area of bark (about 2 cm) was peeled off using a multitool kit for the precise injectional delivery directly into the xylem. The microinjection tip (12 mm long and 1.5 mm thick) was inserted into the tree’s trunk with minimal invasiveness. The trees received treatments according to the specific thesis group. Foliar spray treatment was performed according to the already reported procedure [18]. In the trial period, no fertilization or irrigation was applied to the field. The tree and sample numbers, the date of leaves sample collection, the used treatment and doses are described in Supplementary Material Table S1.

### 4.2. Sample Preparation and NMR Data Processing

Samples of leaves, for ^1^H NMR profiling of xylematic extracts, were collected from the treated trees before every other treatment and analyzed in an overall timespan of 180 days. Samples were collected from the North and South side of the full canopy of each tree at day 0 (before injection) 60, 120, and 180 days after the injection. Each sample was constituted by a statistically representative number of mature leaves (20 leaves) for a total of 60 leaf samples. The samples were harvested and put in labeled plastic bags. They were transported to the laboratory in a chilled box and kept at −20 °C until extraction for metabolomic analysis. The leaf sample extracts for NMR analysis were prepared by applying the experimental procedure previously reported in the literature [49]. In detail: a stainless-steel blender was used to grind into a fine powder the olive leaves (20 leaves for sample) that were preliminarily plunged into liquid N_2_. The crushed leaves were then put into a plastic tube and freeze-dried for 48 h. In a 2 mL Eppendorf tube, 0.75 mL of CD_3_OD and 0.75 mL of KH_2_PO_4_ buffer in D_2_O (pH 5.9) containing 0.05% *w*/*v* TSP-d4 (sodium salt of trimethylsilylpropionic acid) were added to each sample of the lyophilized plant material (100 mg) that was obtained, and extraction was performed. A vortex mixer was used to mix the content of the Eppendorf tubes well for 1 min and then they were sonicated at room temperature for 10 min. The samples were centrifuged at 17,000 g for 20 min; then, a 5 mm NMR tube was filled with 600 µL of the supernatant.

A Bruker Avance III 600 MHz Ascend NMR Spectrometer (Bruker Italia, Milano, Italy) with a TCI cryoprobe (inverse Triple Resonance Cryoprobe Prodigy) with a *z*-axis gradient coil and automatic tuning-matching (ATM) was used to obtain all spectra at 300 K. The operating frequency of the device was 600.13 MHz. Water signal suppression (Bruker pulseprogram zgcppr) was used to acquire the ^1^H NMR spectrum for each sample, in a spectral window of 20.0276 ppm (12,019.230 Hz), 64 scans and a 90° pulse of 7.620 µsec. The standard FID processing was carried out using TopSpin 3.5 (Bruker, Biospin, Italy) after every acquisition. Afterwards, the phase and baseline correction, and 0.3 Hz line broadening was carried out. Amix 3.9.15 (Analysis of Mixture, Bruker BioSpin GmbH, Rheinstetten, Germany) software was used to transform the NMR spectra into a format that was suitable for multivariate analysis. The process involved segmenting each NMR spectrum into regions or histograms with a fixed base width of 0.04 ppm, which is also known as “normal rectangular bucketing”. After that, the data matrices (buckets) were subjected to centering and scaling operations to reduce the variability and noise in the data. The Pareto scaling method was applied, which involved dividing each variable by the square root of the variable standard deviation centered around the mean value [50]. To minimize small differences among the samples that could be caused by metabolites concentration and/or experimental conditions, the total sum normalization was applied. In order to reduce the small differences among the samples due to metabolites concentration and/or experimental conditions the total sum normalization was applied. For further multivariate data analysis, the data table was used that was obtained with all aligned buckets-row reduced NMR spectra. Likewise, every bucket, in a bucket-row for the fragmented spectrum was labeled with the value of the central chemical shift for its specific 0.04 ppm width. The buckets represent the variability of each sample that can be used for statistical analysis.

### 4.3. Multivariate Statistical Analysis Applied to Acquired Data

Following the spectral data processing step, an exploratory and discriminating analysis was carried out with a multivariate statistical approach (MVA), using the SIMCA-P version 14.1 (Sartorius Stedim Biotech, Umeå, Sweden) software, Specifically, Principal Components Analysis (PCA), Partial Least Squares Discriminant Analysis (PLS-DA), and Orthogonal Partial Least Squares Discriminant Analysis (OPLS–DA) were carried out. Unsupervised Principal Component Analysis (PCA) represents the first chemometric technique in data analysis. PCA aims to extract the maximum possible information from a multivariate data structure, reducing it to a few linear combinations of the variables themselves [51].

PCA is commonly used to obtain a general description of the sample distribution and its possible grouping in homogeneous clusters [52]. Supervised methods such as PLS-DA (Projections to Latent Structures Discriminant Analysis) and OPLS–DA (Orthogonal Partial Least Squares Discriminant Analysis) are employed to assess the correlation between the distribution of the potential clusters of the examined samples (seen by PCA) and the classes that are taken into account. For the differentiation of samples with distinct characteristics, the PLS-DA is currently the most commonly used technique. The PLS-DA is carried out by rotating the main components (i.e., the axes that express the variance of the data), to obtain a maximum separation between the defined sample classes, also providing information on the variables responsible for the classes separation [53]. OPLS-DA is a variation of the PLS-DA that eliminates variation not directly related to the focused discriminating response. The isolation of the portion of the variance useful for predictive purposes from the non-predictive variance (which is made orthogonal) is achieved by this technique that can provide the model with enhanced interpretability [54]

The internal cross-validation method and a permutation test, available on the SIMCA-P software, were performed to check the validity and the degree of overfitting for the studied models [55]. The R2 and Q2 parameters were used to characterize the quality of the model. The first (R2) is a cross-validation parameter defined as the explained variance of the models and describes the goodness of fit. The second (Q2) indicates the fraction of the variance in the data predictable by the model [56]. The statistical tool S-line plot was employed to define the variables responsible for the observed discrimination along specific components. The S-line plot is customized for NMR spectroscopy data and generates a plot of the loading vectors, for the considered component, colored according to the correlation scaled loading absolute value, p(corr) [55]. The log2-FC ratio of the standardized median power of corresponding selected bucket-decreased impartial NMR signals after spectra standardization was computed to assess the relative measurement in distinguishing metabolite content between the considered classes The relative quantification in discriminating metabolites between the set classes were evaluated by calculating the log2-FC ratio of the normalized median intensity of corresponding selected bucket-reduced unbiased NMR signals after spectra normalization [57,58].

## 5. Conclusions

The medium-term effects of foliar spray and endo-therapy treatments with different doses of a Cu/Zn citric acid biocomplex (Dentamet^®^) in XFP infected olive trees were studied by ^1^H NMR-based metabolomics of leaf extracts. The current study with foliar spray or endo-therapy (Invaio Sciences TIPS^TM^) with different doses, compared with water treatment (as control), showed a more specific time-related progressive effectiveness of foliar application in comparison to intravascular treatments. Nevertheless, the best results were found for endo-therapy treatments in terms of water injection discrimination. Moreover, persistent endo-therapy (monthly low dose) was much more effective than discontinued treatment (bimonthly high dose) to treat the infected olive tree. The comparisons of the data obtained within the tested endo-therapy conditions suggest the need for further doses/frequencies trimming to obtain long-term results besides a possible more effective performance with respect to foliar treatments. In particular, the resulting data indicates that an improvement in the results could be obtained by optimizing treatments with higher frequencies to reach a possible development toward pathologic remission. In conclusion, this work demonstrates that trunk injection is effective in changing the metabolite profile of the host, possibly modifying the microenvironment within tissues to the point of reducing the ability of the pathogen to create biofilm and proliferate within plant tissues. The obtained results also indicate the importance of a detailed investigation of plant metabolites profiles over subsequent seasons of field experiments for a clear understanding of possible phytotherapy related effects. Notably, the used NMR based metabolomic approach was demonstrated as a powerful tool for the detection of physiological modifications in plant pathology studies and phytotherapeutic protocols fine trimming.

In the present case, the field experiments confirmed that the application of zinc/copper complex (Dentamet^®^) against XFP in infected olive trees results in the reprogramming of specific metabolic pathways with possible positive effects toward disease control. Although a clear curative mechanism for the treatment is not fully understood a possible treatment effect linked with a plant-triggered defense system and/or a direct influence on *X. fastidiosa* by ionic compound accumulation in the vascular bundle could be supposed.

## Data Availability

Data is contained within the article and Supplementary Material.

## Supplementary Materials

The following supporting information can be downloaded at: https://www.mdpi.com/article/10.3390/plants12101946/s1, Table S1: List of the 60 analyzed samples regarding the study of the mid-term effects of the treatment; Table S2: List of the parameters and the figures depicted for all the analyses.
