# Supplementary material for: 1H-NMR Metabolomics Study after Foliar and Endo-Therapy Treatments of Xylella fastidiosa subsp. pauca Infected Olive Trees: Medium Time Monitoring of Field Experiments
Source: Plants (Basel). 2023 May 10;12(10):1946. doi: 10.3390/plants12101946 (PMC10221468; doi:10.3390/plants12101946)
Supplement: Supplementary file 1 [file plants-12-01946-s001.zip › plants-2324408-supplementary.docx]

Supplementary

^1^H-NMR Metabolomics Study After Foliar and Endo-Therapy Treatments of *Xylella fastidiosa* subsp. *pauca* Infected Olive Trees: Medium Time Monitoring of Field Experiments

Mudassar Hussain ^1^, Chiara Roberta Girelli ^1^*, Dimitri Verweire ^2^, Michael C. Oehl ^2^, Maier S. Avendaño ^2^ Marco Scortichini ^3^ and Francesco Paolo Fanizzi ^1^*,

1 Department of Biological and Environmental Sciences and Technologies, University of Salento, Prov.le Lecce-Monteroni, 73100 Lecce, Italy; mudassar.hussain@unisalento.it ; chiara.girelli@unisalento.it ; fp.fanizzi@unisalento.it

2 Invaio Sciences, Cambridge, MA 02138, USA. dverweire@invaio.com; moehl@invaio.com; mavendano@invaio.com

3 Council for Agricultural Research and Agricultural Economic Analyses (CREA) - Research Centre for Olive, Fruit and Citrus Crops, Via di Fioranello, 52, 00134 Roma, Italy; marco.scortichini@crea.gov.it

* Correspondence: chiara.girelli@unisalento.it Tel:+390832299267.; fp.fanizzi@unisalento.it; Tel.: +390832299265

**Table S1. List of the 60 analyzed samples**

| **Sr. No** | **Sample Number** | **Day** | **Treatment Type** | **Treatment Frequency** | **Dosage** | **Tree** | **Date** |
| --- | --- | --- | --- | --- | --- | --- | --- |
| 1 | SAMP39824 | 0 | Water | Monthly | 75ml (w) | 102 | 26/02/2021 |
| 2 | SAMP39825 | 0 | Water | Monthly | 75ml (w) | 205 | 26/02/2021 |
| 3 | SAMP39826 | 0 | Water | Monthly | 75ml (w) | 309 | 26/02/2021 |
| 4 | SAMP39827 | 0 | Foliar | Monthly | 50ml (D) + 14.95l (w) | 108 | 26/02/2021 |
| 5 | SAMP39828 | 0 | Foliar | Monthly | 50ml (D) + 14.95l (w) | 209 | 26/02/2021 |
| 6 | SAMP39829 | 0 | Foliar | Monthly | 50ml (D) + 14.95l (w) | 308 | 26/02/2021 |
| 7 | SAMP39830 | 0 | High Dose | Monthly | 50ml (D) + 100ml (w) | 104 | 26/02/2021 |
| 8 | SAMP39831 | 0 | High Dose | Monthly | 50ml (D) + 100ml (w) | 202 | 26/02/2021 |
| 9 | SAMP39832 | 0 | High Dose | Monthly | 50ml (D) + 100ml (w) | 304 | 26/02/2021 |
| 10 | SAMP39833 | 0 | Low Dose | Monthly | 25ml (D) + 50ml (w) | 103 | 26/02/2021 |
| 11 | SAMP39834 | 0 | Low Dose | Monthly | 25ml (D) + 50ml (w) | 204 | 26/02/2021 |
| 12 | SAMP39835 | 0 | Low Dose | Monthly | 25ml (D) + 50ml (w) | 311 | 26/02/2021 |
| 13 | SAMP39836 | 0 | BiM H.D | Bi-Monthly | 50ml (D) + 100ml (w) | 110 | 26/02/2021 |
| 14 | SAMP39837 | 0 | BiM H.D | Bi-Monthly | 50ml (D) + 100ml (w) | 201 | 26/02/2021 |
| 15 | SAMP39838 | 0 | BiM H.D | Bi-Monthly | 50ml (D) + 100ml (w) | 303 | 26/02/2021 |
| 16 | SAMP44624 | 60 | Water | Monthly | 75ml (w) | 102 | 27/04/2021 |
| 17 | SAMP44625 | 60 | Water | Monthly | 75ml (w) | 205 | 27/04/2021 |
| 18 | SAMP44626 | 60 | Water | Monthly | 75ml (w) | 309 | 27/04/2021 |
| 19 | SAMP44627 | 60 | Foliar | Monthly | 50ml (D) + 14.95l (w) | 108 | 27/04/2021 |
| 20 | SAMP44628 | 60 | Foliar | Monthly | 50ml (D) + 14.95l (w) | 209 | 27/04/2021 |
| 21 | SAMP44629 | 60 | Foliar | Monthly | 50ml (D) + 14.95l (w) | 308 | 27/04/2021 |
| 22 | SAMP44630 | 60 | High Dose | Monthly | 50ml (D) + 100ml (w) | 104 | 27/04/2021 |
| 23 | SAMP44631 | 60 | High Dose | Monthly | 50ml (D) + 100ml (w) | 202 | 27/04/2021 |
| 24 | SAMP44632 | 60 | High Dose | Monthly | 50ml (D) + 100ml (w) | 304 | 27/04/2021 |
| 25 | SAMP44633 | 60 | Low Dose | Monthly | 25ml (D) + 50ml (w) | 103 | 27/04/2021 |
| 26 | SAMP44634 | 60 | Low Dose | Monthly | 25ml (D) + 50ml (w) | 204 | 27/04/2021 |
| 27 | SAMP44635 | 60 | Low Dose | Monthly | 25ml (D) + 50ml (w) | 311 | 27/04/2021 |
| 28 | SAMP44636 | 60 | BiM H.D | Bi-Monthly | 50ml (D) + 100ml (w) | 110 | 27/04/2021 |
| 29 | SAMP44637 | 60 | BiM H.D | Bi-Monthly | 50ml (D) + 100ml (w) | 201 | 27/04/2021 |
| 30 | SAMP44638 | 60 | BiM H.D | Bi-Monthly | 50ml (D) + 100ml (w) | 303 | 27/04/2021 |
| 31 | SAMP44654 | 120 | Water | Monthly | 75ml (w) | 102 | 26/06/2021 |
| 32 | SAMP44655 | 120 | Water | Monthly | 75ml (w) | 205 | 26/06/2021 |
| 33 | SAMP44656 | 120 | Water | Monthly | 75ml (w) | 309 | 26/06/2021 |
| 34 | SAMP44657 | 120 | Foliar | Monthly | 50ml (D) + 14.95l (w) | 108 | 26/06/2021 |
| 35 | SAMP44658 | 120 | Foliar | Monthly | 50ml (D) + 14.95l (w) | 209 | 26/06/2021 |
| 36 | SAMP44659 | 120 | Foliar | Monthly | 50ml (D) + 14.95l (w) | 308 | 26/06/2021 |
| 37 | SAMP44660 | 120 | High Dose | Monthly | 50ml (D) + 100ml (w) | 104 | 26/06/2021 |
| 38 | SAMP44661 | 120 | High Dose | Monthly | 50ml (D) + 100ml (w) | 202 | 26/06/2021 |
| 39 | SAMP44662 | 120 | High Dose | Monthly | 50ml (D) + 100ml (w) | 304 | 26/06/2021 |
| 40 | SAMP44663 | 120 | Low Dose | Monthly | 25ml (D) + 50ml (w) | 103 | 26/06/2021 |
| 41 | SAMP44664 | 120 | Low Dose | Monthly | 25ml (D) + 50ml (w) | 204 | 26/06/2021 |
| 42 | SAMP44665 | 120 | Low Dose | Monthly | 25ml (D) + 50ml (w) | 311 | 26/06/2021 |
| 43 | SAMP44666 | 120 | BiM H.D | Bi-Monthly | 50ml (D) + 100ml (w) | 110 | 26/06/2021 |
| 44 | SAMP44667 | 120 | BiM H.D | Bi-Monthly | 50ml (D) + 100ml (w) | 201 | 26/06/2021 |
| 45 | SAMP44668 | 120 | BiM H.D | Bi-Monthly | 50ml (D) + 100ml (w) | 303 | 26/06/2021 |
| 46 | SAMP44684 | 180 | Water | Monthly | 75ml (w) | 102 | 25/08/2021 |
| 47 | SAMP44685 | 180 | Water | Monthly | 75ml (w) | 205 | 25/08/2021 |
| 48 | SAMP44686 | 180 | Water | Monthly | 75ml (w) | 309 | 25/08/2021 |
| 49 | SAMP44687 | 180 | Foliar | Monthly | 50ml (D) + 14.95l (w) | 108 | 25/08/2021 |
| 50 | SAMP44688 | 180 | Foliar | Monthly | 50ml (D) + 14.95l (w) | 209 | 25/08/2021 |
| 51 | SAMP44689 | 180 | Foliar | Monthly | 50ml (D) + 14.95l (w) | 308 | 25/08/2021 |
| 52 | SAMP44690 | 180 | High Dose | Monthly | 50ml (D) + 100ml (w) | 104 | 25/08/2021 |
| 53 | SAMP44691 | 180 | High Dose | Monthly | 50ml (D) + 100ml (w) | 202 | 25/08/2021 |
| 54 | SAMP44692 | 180 | High Dose | Monthly | 50ml (D) + 100ml (w) | 304 | 25/08/2021 |
| 55 | SAMP44693 | 180 | Low Dose | Monthly | 25ml (D) + 50ml (w) | 103 | 25/08/2021 |
| 56 | SAMP44694 | 180 | Low Dose | Monthly | 25ml (D) + 50ml (w) | 204 | 25/08/2021 |
| 57 | SAMP44695 | 180 | Low Dose | Monthly | 25ml (D) + 50ml (w) | 311 | 25/08/2021 |
| 58 | SAMP44696 | 180 | BiM H.D | Bi-Monthly | 50ml (D) + 100ml (w) | 110 | 25/08/2021 |
| 59 | SAMP44697 | 180 | BiM H.D | Bi-Monthly | 50ml (D) + 100ml (w) | 201 | 25/08/2021 |
| 60 | SAMP44698 | 180 | BiM H.D | Bi-Monthly | 50ml (D) + 100ml (w) | 303 | 25/08/2021 |

*Dentamet® quantity denoted by (D) and water with (W).

**Table S2. List of the parameters and the figures depicted for all the analyses.**

| **Fig. No.** | **Model** | **Plot** | **Components** | **R2X** | **R2Y** | **Q2** |
| --- | --- | --- | --- | --- | --- | --- |
| 1 | PCA | All day class IDs | 4 | 0.783 | - | 0.68 |
| 2 | PCA | All treatment class IDs | 4 | 0.783 | - | 0.68 |
| 3 | PLS-DA | Supervised analysis of all samples | 4 | 0.709 | 0.689 | 0.566 |
| 4 | OPLS-DA | T0 vs all treated | 1+1+0 | 0.504 | 0.839 | 0.805 |
| 5 | OPLS-DA | Agrochemicals vs water treatments | 1+1+0 | 0.463 | 0.225 | 0.0347 |
| 6 | OPLS-DA | Foliar treated vs water, day 60 | 1+1+0 | 0.838 | 0.727 | 0.232 |
| 7 | OPLS-DA | Foliar treated vs water, day 120 | 1+1+0 | 0.834 | 0.857 | 0.445 |
| 8 | OPLS-DA | Foliar treated vs water, day 180 | 1+1+0 | 0.661 | 0.97 | 0.467 |
| 9 | OPLS-DA | Low dose vs water treated, day 60 | 1+1+0 | 0.774 | 0.89 | -0.57 |
| 10 | OPLS-DA | Low dose vs water treated, day 120 | 1+1+0 | 0.731 | 0.982 | 0.621 |
| 11 | OPLS-DA | Low dose vs water treated, day 180 | 1+1+0 | 0.222 | 0.982 | 0.103 |
| 12 | OPLS-DA | High dose vs water treated, day 60 | 1+1+0 | 0.738 | 0.831 | -0.053 |
| 13 | OPLS-DA | High dose vs water treated, day 120 | 1+1+0 | 0.848 | 0.781 | 0.551 |
| 14 | OPLS-DA | High dose vs water treated, day 180 | 1+1+0 | 0.514 | 0.926 | 0.47 |
| 15 | OPLS-DA | Bimonthly high dose vs water, day 60 | 1+1+0 | 0.753 | 0.726 | -0.45 |
| 16 | OPLS-DA | Bimonthly high dose vs water, day 120 | 1+1+0 | 0.726 | 0.881 | 0.366 |
| 17 | OPLS-DA | Bimonthly high dose vs water, day 180 | 1+1+0 | 0.644 | 0.87 | 0.16 |
| 18 | Bar chart | Dentamet vs water treatments | - | - | - | - |
| 19 | FC chart | Metabolites’ fold change | - | - | - | - |
| 20 | FC chart | Comparison of fold changes | - | - | - | - |
